# Effect of Gabapentin on morphine demand and pain after laparoscopic sterilization using Filshie clips. A double blind randomized clinical trial

**DOI:** 10.1186/1471-2253-6-12

**Published:** 2006-11-03

**Authors:** Jens Bartholdy, Karen L Hilsted, Nils C Hjortsoe, Jens Engbaek, Joergen B Dahl

**Affiliations:** 1 Department of Day Surgery, Copenhagen University Hospital Herlev, Herlev Ringvej 75 2730 Herlev, Denmark; 2 Department of Anaesthesiology, Copenhagen University Hospital Glostrup, Nordre Ringvej 57 2600 Glostrup, Denmark; 3 Department of Day Surgery, Copenhagen University Hospital Glostrup, Nordre Ringvej 57 2600 Glostrup, Denmark

## Abstract

**Background:**

A considerable number of patients require opioids during recovery after laparoscopic sterilization. This implies nausea, dizziness and sedation and increases the number of unplanned admissions. Gabapentin has shown excellent postoperative analgesic effect in a number of recent studies with few side effects. This study was designed to test whether gabapentin given preoperatively can reduce the number of patients needing morphine in the recovery period.

**Methods:**

80 females scheduled for laparoscopic sterilization using Filshie clips were randomized to two treatment groups (Gaba group and control group).

All patients received lornoxicam 8 mg p.o. 30 min. before the procedure. Patients in the Gaba group received gabapentin 1200 mg p.o. and patients in the control group received placebo capsules prior to the procedure. All patients were anesthetized according to a protocol, using remifentanil and propofol.

Postoperative analgesia was obtained with patient controlled infusion of morphine. Pain, nausea, dizziness and sedation were scored at 2 and 4 hours after end of anesthesia. The expenditure of morphine was the primary measure for the effect of analgesia and the number of patients demanding morphine was the primary endpoint.

**Results:**

Three patients were excluded because of procedural errors and one because of conversion to open surgery. 38 patients completed the study in each group.

32 (84%) patients in the gabapentin group and 37 (97%) patients in the control group did require morphine in the recovery period. (p = 0,049).

There was no significant difference between mean morphine consumption, pain scores and frequency of adverse effects (nausea, dizziness, sedation and vomiting)

**Conclusion:**

The postoperative analgesic effect of gabapentin given preoperatively was confirmed in this study. For this procedure, with pain predominantly in the immediate recovery period, and of less intensity than after major surgical procedures, the effect demonstrated is much less pronounced than in similar studies of major surgery. General use of gabapentin as analgesic for laparoscopic sterilization is not supported by this study.

**Trial Registration:**

Current Controlled Trials ISCRTN39209275

## Background

Laparoscopic sterilization of females is a procedure well suited for day surgery. A considerable number of patients however, require opioids in the recovery period [1], delaying the time for street fitness and implying a number of unplanned admissions due to nausea, dizziness and sedation.

An analgesic regimen with less adverse effects is thus desirable.

Recently, gabapentin has been introduced as an adjunctive analgesic drug in the perioperative setting, and results so far are promising [2-8]. A number of meta-analyses have been conducted showing reduced pain and opioid requirements when gabapentin was administered preoperatively [9-12]. Although data so far are insufficient for firm conclusions, the most recent meta-analysis demonstrated both reduced pain and opioid requirements as well as less opioid-related side effects such as vomiting and pruritus [12].

This study was designed to test whether gabapentin combined with the NSAID lornoxicam given preoperatively can reduce the need for postoperative morphine compared with preoperative lornoxicam alone in patients scheduled for laparoscopic sterilization.

## Methods

Women aged 26–45 yr scheduled for laparoscopic sterilization using Filshie clips were eligible for the study. Patients were not included if they were unable to cooperate, were breast feeding, had known allergy to gabapentin or morphine, a history of drug or alcohol abuse, chronic pain or daily intake of analgesics or corticosteroids, diabetes, or impaired kidney function. Patients with an intake of NSAIDs or paracetamol 24 h prior to operation or an intake of antacids 48 h prior to operation were also excluded from the study. Patients were recruited from the Day Surgical Departments of Copenhagen University Hospital, Herlev (Herlev, Denmark) and Copenhagen University Hospital, Glostrup (Glostrup, Denmark) during the period September 2002 to November 2004. Written informed consent was obtained from all patients, and the study was approved by the Regional Ethics Committee (Herlev, Denmark) and The Danish Medicine Agency (Copenhagen, Denmark).

### Interventions

Patients received oral lornoxicam 8 mg, combined with oral gabapentin 1.200 mg or placebo, 30 min before surgery.

General anesthesia was induced with 1.5–2.5 mg/kg propofol, and infusion of 1 μg/kg remifentanil for 1 min.

A pro seal laryngeal mask (LMA-PS) was inserted. A nasogastric tube was introduced through the LMA-PS to drain the stomach of air. Liquid gastric content was returned through the nasogastric tube. Anesthesia was maintained with infusion of propofol at the discretion of the anesthetist, and a fixed infusion of 0.4 μg·kg^-1 ^min^-1 ^remifentanil. Hypotension was treated with 5 mg ephedrine intravenously in incremental doses, in order to preserve systolic blood pressure above 90 mmHg. The infusions of propofol and remifentanil were terminated at skin closure; 0.5 mg alfentanil was administered intravenously to all patients, who were then transferred to the postoperative care unit. Postoperative pain treatment consisted of patient-controlled intravenous morphine (Abbott Pain Management Provider; Abbott, Virum, Denmark). Initial bolus dose was 5 mg, supplemental bolus doses were 2.5 mg. Lock-out time was 10 min. Additional morphine, 2.5 mg intravenously, was administered by a nurse observer, if requested by the patient, during the lock-out period. Ondansetron, 4 mg intravenously, was administered on patient request. No other medications were administered during the 4-h observation period.

### Outcomes and assessments

The primary outcome measure was number of patients requesting morphine during the first 4 postoperative hours.

Secondary outcome measures were: Total morphine consumption from 0 to 4 h postoperatively; pain at rest and during mobilization from the supine to the sitting position, and side effects: nausea, sedation, dizziness, and vomiting.

Before surgery, all patients were instructed in the use of patient-controlled analgesia (PCA) and the visual analogue score (VAS) (0 mm: no pain, 100 mm: worst pain imaginable). Total morphine consumption was recorded from 0 to 4 h postoperatively. Pain scores at rest and during mobilization were assessed by the patients at 2 and 4 h after surgery.

Side effects were rated on a four-point verbal scale (none, mild, moderate, severe) at 2 and 4 h after surgery.

The number of patients vomiting, as well as use of antiemetics, was recorded.

### Study population size

Based on registration of morphine administration for this procedure during the year before the study 60 % of patients had morphine in the postoperative period after laparoscopic sterilization. We considered a 30 % reduction in this frequency to be clinically relevant. With a type 1 error of 5 % and a power of 90% 38 patients were required in each study group.

### Blinding

The study was randomized, double-blind, and placebo controlled. Study medication was prepared by the hospital pharmacy into identical capsules containing either 300 mg gabapentin, or placebo. Study medication was marked with the name of the project, the investigator's name, and consecutive numbers according to a computer-generated block randomization schedule prepared by the hospital pharmacy. Patients were enrolled by the same investigators who also performed the assessments. Participants were assigned consecutively to their group according to their number. No person was aware of group assignment until all patients had been included and assessments were completed.

### Statistical methods

The Kolmogorov-Smirnov one-sample test for normality (K-S) was performed on the data sets to examine if t-test was possible. The K-S test was significant for all data sets except total morphine consumption. Consequently total morphine consumption was compared using Students t-test, and all other variables were compared using Mann-Whitney rank sum test for unpaired data.

Bonferronis correction was used for multiple comparisons.

Data are presented as medians with lower and upper quartiles. Calculations were performed using SPSS 13.0 for Windows (SPPS, Chicago, IL). The statistical analysis was performed by the investigators.

## Results

**Figure 1:**
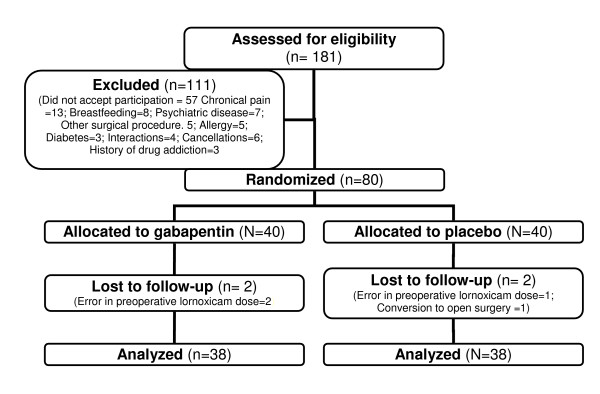
Flow diagram of patient distribution.

181 consecutive patients who fulfilled the inclusion criteria were considered for inclusion in the study (Fig. 1). Eighty patients were included in the study; four of these were subsequently excluded, two in the gabapentin and two in the placebo group. Three patients were given doses of lornoxicam preoperatively other than prescribed in the study protocol, two in the placebo group and one in the gabapentin group. One procedure in the gabapentin group was conversed to open surgery during the operation.

Data from 76 patients, 38/40 in the gabapentin group and 38/40 in the placebo group, were analyzed.

Baseline demographic and clinical characteristics of each group are shown in Table 1. No significant differences were observed between groups.

**Table 1:** Demographics

	Gabapentin	Control
**Number of patients (n)**	38	38
**Age**	37 (28–45)	38 (27–45)
**Height (cm)**	165 (151–184)	168 (154–179)
**Weight (kg)**	65 (47–110)	64 (49–110)
**Duration of surgery (min)**	34 (16–77)	29 (14–65)

Patient and perioperative data (median and range).

No significant differences between groups.

### Number of patients requesting morphine

Thirty-two of 38 patients in the gabapentin group and 37/38 patients in the control group did require morphine in the postoperative period. The difference is significant (p = 0.049)

### Morphine consumption

No statistically significant difference in the median amount of morphine required during the first 4 postoperative hours was found (table 2).

**Table 2:** Morphine consumption:

	**Gabapentin (n = 38)**	**Control (n = 38)**	**P**
**Number of patients consuming morphine postoperatively**	32 (84%)	37 (97%)	0.049^1^
**Total consumption of morphine in mg** **(mean ± st. dev)**	10.5 ± 7,1	13.7 ± 7,4	0.059^2^

Frequency of demand for morphine (PCA) postoperatively and mean consumption of morphine (PCA) postoperatively. (1: Mann-Witney; 2: Student t)

### Pain scores

No significant differences between pain scores at rest or during mobilization were found at any assessment (Table 3).

**Table 3:** Pain scores

	**Gabapentin (n = 38)**	Control (n = 38)	**P (Mann-witney)**
**VAS at rest 2 hours after operation**	13 (7–28)	22.5 (11–35)	0.06
**VAS at rest 4 hours after operation**	4 (0–10)	6 (1–11)	0.22
**VAS sitting up 2 hours after operation**	8 (4.5–26)	20(6.5–29)	0.26
**VAS sitting up 4 hours after operation**	3 (0–10)	5 (1–14)	0.49

Pain scores (VAS median, lower and upper quartile) at rest and when urged to move from recumbent to sitting position.

### Side-effects

The incidence of side-effects appears from Table 4. No significant differences were observed in any outcome between groups (P > 0.05 for all observations).

**Table 4:** Side effects

**Side effect**	**2 h postoperatively**		**4 h postoperatively**	
	**Control (n = 38)**	**Gabapentin (n = 38)**	**Control (n = 38)**	**Gabapentin (n = 38)**
**Nausea**				
None/Mild	36	38	34	35
Moderate/Severe	2	0	4	3

**Sedation**				
None/Mild	15	19	25	17
Moderate/Severe	23	19	13	21

**Dizziness**				
None/Mild	29	28	28	26
Moderate/Severe	9	10	10	12

**Vomiting**				
1 time or more			3	3

No significant difference between groups.

## Discussion

This study did show a significant reduction in the number of patients requiring morphine after laparoscopic sterilization. (The primary outcome parameter). In contrast to other studies, the reduction in mean amount of morphine used was small and not statistically significant. The clinical implication is that gabapentin is not likely to be of use in this setting.

The only earlier study of the effect of gabapentin on postoperative pain after a laparoscopic procedure did show a marked effect of a small dose of gabapentin given preoperatively on pain and opioid consumption after laparoscopic cholecystectomy [13]. In this study no additional analgesic was given beside the "rescue" drug (fentanyl).

The sensitivity of the current study was reduced by the design, where both groups did receive NSAID (lornoxicam). This study does actually compare the use of gabapentin versus placebo as an additive to NSAID for pain after sterilization. This was done for ethical reasons, as preoperative NSAID is routinely used for this procedure in our institutions. The efficacy of NSAID per se in postoperative pain control after laparoscopic sterilization is difficult to estimate based on the literature, as CRT's give result ranging from non significant effect to elimination of the need for morphine [1,14-19]. In our institutions data from the local quality assurance database did show that 60% of the patients did require postoperative opioid administration despite routine preoperative administration of NSAID.

Another factor diminishing the sensitivity of this study to demonstrate analgesic effect of gabapentin might be the short interval between the administration of gabapentin and the end of surgery. Gabapentin concentrations might not have reached optimal levels in all cases in the immediate recovery period. However: To be useful in day surgery, a drug has to be active within an hour after administration so the design reflects the clinical reality.

The technique of using the consumption of morphine during PCA treatment of postoperative pain, as a measure of the effect of the analgesic regime under study, has been used in several other studies of this kind [5,6]. Due to the delayed onset of the analgesic effect of morphine, titration for optimal pain control might be rather difficult for the patient in the immediate recovery period. This might be a minor drawback, when a postoperative analgesic regimen for major surgery is the object of study, but it diminishes the sensitivity of the technique severely, when the period of severe pain, is limited to few hours postoperatively. In future studies in the setting of day surgery, it might be appropriate to use opioids with a shorter onset time and time of action than morphine.

The frequency of side effects was not different between the groups in this study. However the study was not dimensioned to disclose such a difference, and a type 2 error in these the results is quite likely. A non significant trend in the results with a slightly higher frequency of dizziness in the gabapentin group and a slightly higher frequency of nausea in the control group is noted. This is in accordance with earlier studies in the ambulatory surgical setting [3]

## Conclusion

The postoperative analgesic effect of gabapentin given preoperatively was confirmed in this study. For this procedure, with pain predominantly in the immediate recovery period, and of less intensity than after major surgical procedures, the effect demonstrated is much less pronounced than in similar studies of major surgery. General use of gabapentin as analgesic for laparoscopic sterilization is not supported by this study.

## Competing interests

JBD has received an unrestricted research grant from Pfizer Denmark

## Authors' contributions

JB participated in the design of the study recruited patients and carried out most of the anaesthesias and drafted the manuscript. JE and NCH participated in the design of the study recruited a number of patients, carried out some anaesthesias and helped prepare the manuscript. JBD and KHL conceived of the study, and participated in its design and coordination and helped to draft the manuscript. All authors read and approved the final manuscript.

## Pre-publication history

The pre-publication history for this paper can be accessed here:



## References

[B1] Alexander JI (1997). Pain after laparoscopy. Br J Anaesth.

[B2] Pandey CK, Sahay S, Gupta D, Ambesh SP, Singh RB, Raza M, Singh U, Singh PK (2004). Preemptive gabapentin decreases postoperative pain after lumbar discoidectomy. Can J Anaesth.

[B3] Turan A, Memis D, Karamanlioglu B, Yagiz R, Pamukcu Z, Yavuz E (2004). The analgesic effects of gabapentin in monitored anesthesia care for ear-nose-throat surgery. Anesth Analg.

[B4] Turan A, Karamanlioglu B, Memis D, Usar P, Pamukcu Z, Ture M (2004). The analgesic effects of gabapentin after total abdominal hysterectomy. Anesth Analg.

[B5] Dierking G, Duedahl TH, Rasmussen ML, Fomsgaard JS, Moiniche S, Romsing J, Dahl JB (2004). Effects of gabapentin on postoperative morphine consumption and pain after abdominal hysterectomy: a randomized, double-blind trial. Acta Anaesthesiol Scand.

[B6] Dirks J, Fredensborg BB, Christensen D, Fomsgaard JS, Flyger H, Dahl JB (2002). A randomized study of the effects of single-dose gabapentin versus placebo on postoperative pain and morphine consumption after mastectomy. Anesthesiology.

[B7] Fassoulaki A, Stamatakis E, Petropoulos G, Siafaka I, Hassiakos D, Sarantopoulos C (2006). Gabapentin attenuates late but not acute pain after abdominal hysterectomy. Eur J Anaesthesiol.

[B8] Gilron I, Orr E, Tu D, O'Neill JP, Zamora JE, Bell AC (2005). A placebo-controlled randomized clinical trial of perioperative administration of gabapentin, rofecoxib and their combination for spontaneous and movement-evoked pain after abdominal hysterectomy. Pain.

[B9] Hurley RW, Cohen SP, Williams KA, Rowlingson AJ, Wu CL (2006). The analgesic effects of perioperative gabapentin on postoperative pain: a meta-analysis. Reg Anesth Pain Med.

[B10] Gilron I (2006). Review article: the role of anticonvulsant drugs in postoperative pain management: a bench-to-bedside perspective. Can J Anaesth.

[B11] Seib RK, Paul JE (2006). Preoperative gabapentin for postoperative analgesia: a meta-analysis. Can J Anaesth.

[B12] Ho KY, Gan TJ, Habib AS (2006). Gabapentin and postoperative pain - a systematic review of randomized controlled trials. Pain.

[B13] Pandey CK, Priye S, Singh S, Singh U, Singh RB, Singh PK (2004). Preemptive use of gabapentin significantly decreases postoperative pain and rescue analgesic requirements in laparoscopic cholecystectomy. Can J Anaesth.

[B14] Green CR, Pandit SK, Levy L, Kothary SP, Tait AR, Schork MA (1996). Intraoperative ketorolac has an opioid-sparing effect in women after diagnostic laparoscopy but not after laparoscopic tubal ligation. Anesth Analg.

[B15] White PF, Joshi GP, Carpenter RL, Fragen RJ (1997). A comparison of oral ketorolac and hydrocodone-acetaminophen for analgesia after ambulatory surgery: arthroscopy versus laparoscopic tubal ligation. Anesth Analg.

[B16] Tool AL, Kammerer-Doak DN, Nguyen CM, Cousin MO, Charsley M (1997). Postoperative pain relief following laparoscopic tubal sterilization with silastic bands. Obstet Gynecol.

[B17] Ng A, Temple A, Smith G, Emembolu J (2004). Early analgesic effects of parecoxib versus ketorolac following laparoscopic sterilization: a randomized controlled trial. Br J Anaesth.

[B18] Dahl JB, Mathiesen O, Moiniche S (2004). 'Protective premedication': an option with gabapentin and related drugs? A review of gabapentin and pregabalin in in the treatment of post-operative pain. Acta Anaesthesiol Scand.

[B19] Williams PI (1996). Naproxen after day-case laparoscopic sterilization. Br J Anaesth.

